# A two-gene random forest model to diagnose osteoarthritis based on RNA-binding protein-related genes in knee cartilage tissue

**DOI:** 10.18632/aging.204469

**Published:** 2023-01-05

**Authors:** Wenhua Yin, Ying Lei, Xuan Yang, Jiawei Zou

**Affiliations:** 1 Department of Orthopaedics, Yuebei People’s Hospital Affiliated to Medical College of Shantou University, Shaoguan, Guangdong 512026, China; 2 Department of Audit, Yuebei People’s Hospital Affiliated to Medical College of Shantou University, Shaoguan, Guangdong 512026, China

**Keywords:** osteoarthritis, RNA-binding protein, diagnosis, machine learning, random forest

## Abstract

Osteoarthritis (OA) is one of the most common diseases in the orthopedic clinic, characterized by progressive cartilage degradation. RNA-binding proteins (RBPs) are capable of binding to RNAs at transcription and translation levels, playing an important role in the pathogenesis of OA. This study aims to investigate the diagnosis values of RBP-related genes in OA. The RBPs were collected from previous studies, and the GSE114007 dataset (control = 18, OA = 20) was downloaded from the Gene Expression Omnibus (GEO) as the training cohort. Through various bioinformatical and machine learning methods, including genomic difference detection, protein-protein interaction network analyses, Lasso regression, univariate logistic regression, Boruta algorithm, and SVM-RFE, RNMT and RBM24 were identified and then included into the random forest (RF) diagnosis model. GSE117999 dataset (control = 10, OA = 10) and clinical samples collected from local hospital (control = 10, OA = 11) were used for external validation. The RF model was a promising tool to diagnose OA in the training dataset (area under curve [AUC] = 1.000, 95% confidence interval [CI] = 1.000-1.000), the GSE117999 cohort (AUC = 0.900, 95% CI = 0.769–1.000), and local samples (AUC = 0.759, 95% CI = 0.568–0.951). Besides, qPCR and Western Blotting experiments showed that RNMT (*P* < 0.05) and RBM24 (*P* < 0.01) were both down-regulated in CHON-001 cells with IL-1β treatment. In all, an RF model to diagnose OA based on RNMT and RBM24 in cartilage tissue was constructed, providing a promising clinical tool and possible cut-in points in molecular mechanism clarification.

## INTRODUCTION

As one of the most common diseases worldwide, osteoarthritis (OA) severely affects the life quality of patients and brings a tremendous socioeconomic burden [1]. The pathogenic factors of OA mainly include the alternation of genetics, metabolism, inflammation, and biomechanics, contributing to cartilage destruction and bone fragmentation [2]. However, given the fact that there is no cure for OA with the existing medical treatment except for artificial joint replacement [3], investigating the latent molecular mechanisms to better clarify the etiology and pathogenesis and to develop novel therapeutic agents is urgently demanded.

RNA-binding protein (RBP) is defined as a group of proteins that are able to distinguish and bind to the target RNAs and thus influent these RNAs’ transcription, editing, splicing, and other biological processes [4]. RBPs are vital to maintaining body homeostasis, and the disorders of RBPs lead to a series of diseases, such as inflammatory diseases [5], metabolism-related diseases [6], malignant cancer [7], and genetic diseases [8]. Similar to these situations, RBPs are also involved in the pathological processes of OA. For instance, the RBP PUM1 could alleviate the cartilage destruction caused by OA through down-regulating TLR4 [9]; CPEB1, an RBP binding to the 3′-UTR-cytoplasmic polyadenylation element of the target RNAs, was up-regulated in the cartilage samples of OA patients, and was associated with the severity of the disease [10]; the lack of the RBP Samd4 could lead to the chondrogenesis defects in mice [11]. This evidence proves that RBPs exert non-negligible functions in OA. Nevertheless, our understandings of the roles of RBPs in the initiation and development of OA are far from enough, and the associated researches are at the initial stage for the moment. Seeking more RBPs serving as biomarkers in OA is important and meaningful.

Herein, the present study downloaded the transcriptome sequencing data of the knee cartilage tissue extracted from OA patients from the Gene Expression Omnibus (GEO) since knee OA is the most common form of arthritis. Diverse bioinformatical methods and machine learning algorithms, including genomic difference detection, protein-protein interaction (PPI) network analyses, Lasso regression, univariate logistic regression, Boruta algorithm, and SVM-RFE, were used for feature selection. Then, a random forest (RF) model was developed to diagnose OA. 21 clinical samples from the local hospital and the GSE117999 dataset from GEO were used for external validation. At last, we established an *in vitro* OA model in CHON-001 cells using Interleukin-1β (IL-1β) and measured the expression levels of the screened genes via real-time quantitative PCR (RT-qPCR) and Western Blotting.

## MATERIALS AND METHODS

### Data collection and processing

1525 RBPs identified by experimental research and high-throughput screening were collected from Stefanie and his colleagues’ report [12]. GSE114007 [13], which contained the RNA sequencing (RNA-seq) data of the knee cartilage tissue isolated from 18 healthy control and 20 OA subjects, was downloaded from GEO (https://www.ncbi.nlm.nih.gov/geo/) as the training dataset. The GSE117999 dataset, which was also obtained from GEO and included the transcriptome data of the knee cartilage tissue extracted from 10 control and 10 OA cases, was set as the external validation dataset. To reduce the divergence as possible, we transformed the RNA-seq data into Transcripts-per-Million (TPM) format and then used the sva package in R software (version 3.6.3) to correct the batch effects. The detailed information of these public datasets is displayed in Table 1.

**Table 1:** The detailed information of the public datasets obtained from GEO.

**GEO series**	**Platform**	**Experiment type**	**Tissue**	**Control/OA**	**Region**
GSE114007	GPL11154, GPL18573	RNA-seq	Human knee cartilage	18/20	USA
GSE117999	GPL20844	Microarray	Human knee cartilage	10/10	USA

Abbreviations: GEO: Gene Expression Omnibus; OA: Osteoarthritis.

### Ethics and clinical specimens

The protocol of the present study has been revised and approved by the Medical Ethics Committee of Yuebei People’s Hospital Affiliated to Medical College of Shantou University. All the participants have signed the informed consent. The knee cartilage samples were collected from 11 OA patients (4 female, 7 male, age range 49–73 years) undergoing total knee replacement and 10 subjects (6 female, 4 male, age range 52–71 years) receiving traumatic amputation in the absence of rheumatoid arthritis or OA in Yuebei People’s Hospital Affiliated to Medical College of Shantou University between January 12, 2022 and July 28, 2022. The cartilage tissue was then immediately stored in liquid nitrogen for RNA extraction. The diagnosis of OA depends on the criteria recommended by the American College of Rheumatology.

### Genomic difference analyses

The limma package in R software was used for genomic divergence detection. The false discovery rate (FDR) < 0.05 and |logFC| > 1 were set as the filtering thresholds.

### Gene functional annotation

The gene functional annotation was conducted via the Metascape database [14] (https://metascape.org/) or the clusterProfile package in R. The terms with *P* < 0.05 were considered to be significant.

### PPI network construction

The STRING database (https://cn.string-db.org/) was utilized to construct the PPI network with a confidence level of 0.4. Subsequently, the Cytoscape software (version 3.8.0) was used to visualize the network. The importance of the genes in the network was measured by the cytoHubba plug-in [15], which is a widely-used tool to calculate node scores according to various algorithms including MCC, DMNC, Degree, EPC, Bottle neck, EcCentricity, closeness, radiality, betweenness, stress, and clustering coefficient. Here, we chose “Degree” algorithm to measure the importance of the genes, and the Top 20 genes with the highest degree were included in further analysis.

### Feature selection via machine learning

To identify the genes significantly associated with OA, we implemented various machine learning algorithms, as previously reported [16]. Lasso regression with 10-fold cross-validation was conducted via the glmnet package. The Boruta algorithm was conducted by the Boruta package, and the features labeled with “Confirmed” were selected. The caret package was used to conduct the SVM-RFE. Last, univariate logistic regression was performed with the rms package, where *P* < 0.01 was statistically significant.

### RF model construction

Compared with the conventional modeling method, RF attracted increasing attention due to its high precision and accuracy [17, 18]. Here, we attempted to construct the diagnosis model via RF. The randomForest package in R software was used to develop the RF diagnosis model with ntree = 500 and mtry = 3. The importance of the genes in the RF model was quantified by mean decrease accuracy and mean decrease Gini [16].

### Functional co-expression analyses

The Top 20 functionally co-expressed genes of the target gene were queried in the GeneMANIA database (http://genemania.org/) with the Max resultant genes = 20 and the Max resultant attributes = 10. The interaction types included physical interactions, co-expression, prediction, co-localization, genetic interactions, pathways, and shared protein domains. Subsequently, the functional enrichment of these genes was investigated via the clusterProfile package, where Gene Ontology (GO) datasets were selected as the reference.

### Cell culture and treatment

CHON-001 cell line was obtained from the American Type Culture Collection (Manassas, VA, USA) and cultured in DMEM (Gibco; Thermo Fisher Scientific, USA) supplemented with 10% FBS (Thermo Fisher Scientific, USA) and 1% penicillin-streptomycin at 37°C with 5% CO_2_. CHON-001 cells were incubated with 10 ng/mL IL-1β (Sigma-Aldrich, China) for 48 hours to establish the OA cell model [19, 20].

### RT-qPCR

The total RNA of the clinical samples and cells was extracted with the TRIzol reagent (Invitrogen, USA). The cDNA was synthesized by PrimeScript RT Reagent Kit (Takara, China), and the qPCR experiments were conducted via SYBR Premix ExTaq kit (Takara, China). GAPDH was chosen as the internal reference gene, and the 2^−ΔΔCt^ method was used to normalize the gene expression levels. The primer sequence of this study is shown in Table 2.

**Table 2:** The primer sequence used in this study.

**Gene**	**Sequence (5′-3′)**
RBM24	F: GAACCTGGCATACTTAGGAGCA
	R: AGGTCTTTGTATAAGGGCTGGA
RNMT	F: ATAGCACTTGAGGATGTTCCTGA
	R: ACTACGCTTCTCCAAACCAAC
GAPDH	F: GGAGCGAGATCCCTCCAAAAT
	R: GGCTGTTGTCATACTTCTCATGG

### Western blotting

The cell and tissue samples were lysed with RIPA lysis buffer (Thermo Fisher Scientific, USA) with protease inhibitor (Sigma-Aldrich, USA) on ice. The proteins were transferred onto nitrocellulose membranes (Millipore, USA) after the separation on SDS-PAGE. The membranes were washed using TBS 5 times and then blocked via 5% skimmed milk for 1 hour. Subsequently, the membranes were incubated with primary antibodies overnight at 4°C. Antibodies used in this study: GAPDH (dilution: 1:1000, AC001, ABclonal, China), RNMT (dilution: 1:500, PA5-41778, Thermo Fisher Scientific, USA), and RBM24 (dilution: 1:500, PA5-66881, Thermo Fisher Scientific, USA). Subsequently, the membranes were incubated with secondary anti-rabbit IgG antibodies for 1 hour. MiniChmei Chemiluminescence imager (Sagecreation, China) was used to quantify the protein levels.

### Statistical analyses

The statistical analyses of the present study were based on R software (version 3.6.3) and GraphPad Prism 8 (version 8.4.3). The receiver operating characteristic (ROC) curves were plotted and the areas under the curve (AUCs) were calculated via the pROC package. The gene expression levels detected via RT-qPCR were compared with the student’s *t*-test. Unless otherwise specified, *P* < 0.05 was considered to be statistically significant. ^*^P < 0.05; ^**^P < 0.01; ^***^P < 0.001.

## RESULTS

### Identification of the differentially-expressed RBPs

**Figure 1:**
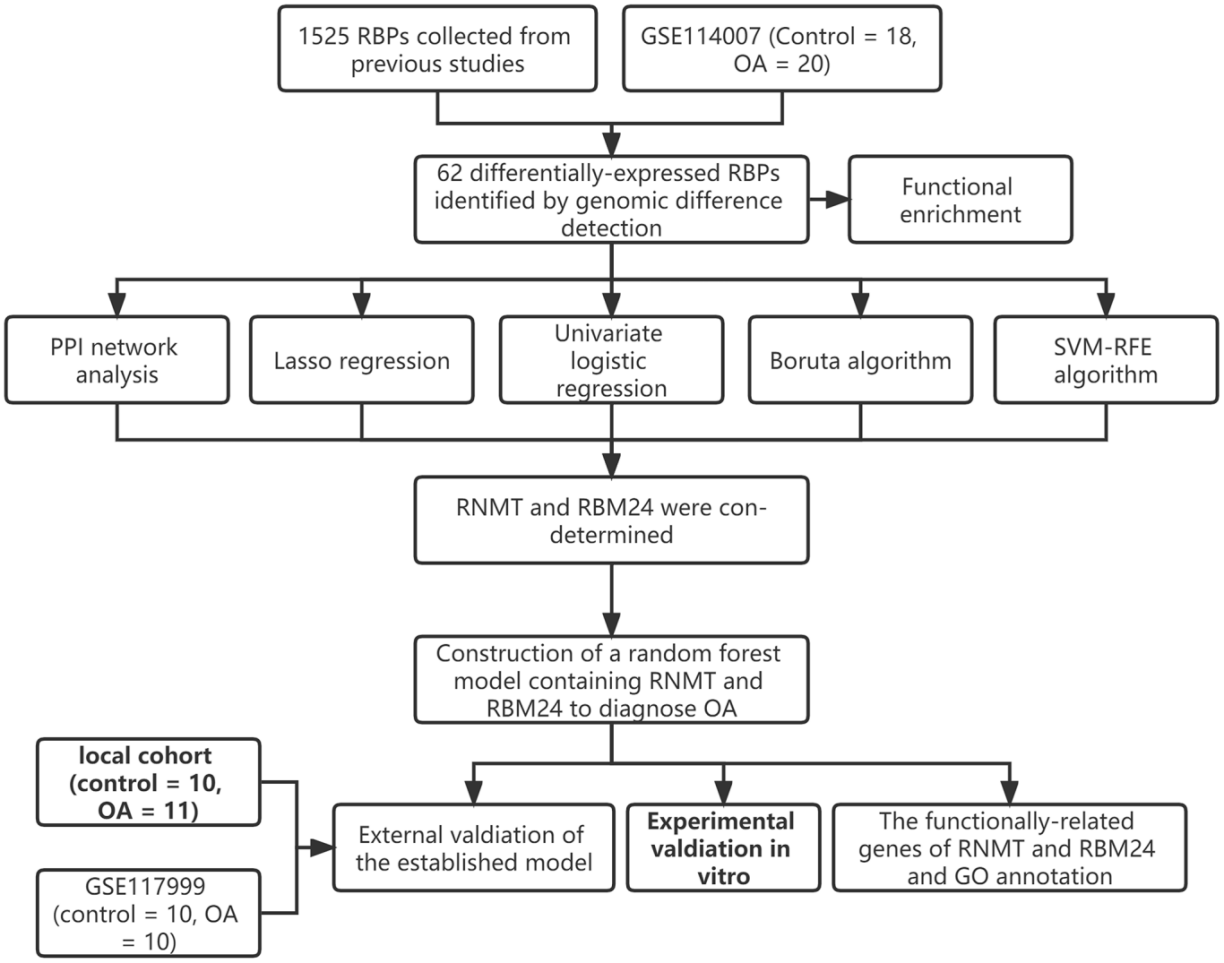
The workflow of the present study.

**Figure 2:**
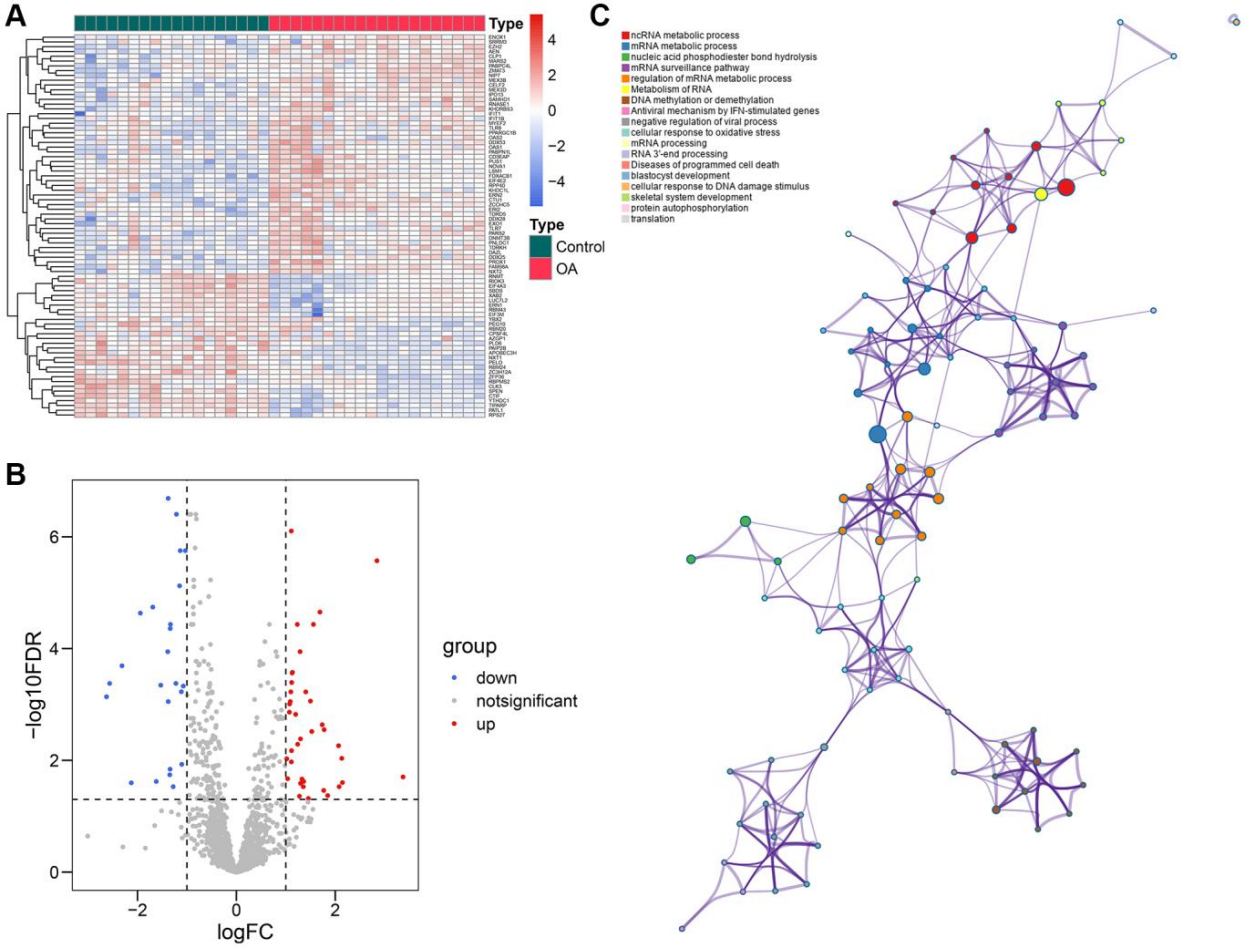
**Identification of the differentially-expressed RBPs.** (**A**, **B**) The volcano plot (**A**) and the heatmap (**B**) showed that a total of 62 differentially-expressed RBPs were detected between the control and OA samples. (**C**) The functional annotation of the 62 genes via the Metascape database. Abbreviations: RBP: RNA-binding protein; OA: osteoarthritis.

The workflow of this study is illustrated in Figure 1, and the R codes used in this study were shown in Supplementary File 1. After the genomic difference analysis, a sum of 62 differentially-expressed RBPs were identified between the control and OA samples of the GSE114007 cohort, including 38 up-regulated and 24 down-regulated genes (Supplementary Table 1). The heatmap and the volcano plot used to visualize the genomic difference detection results were displayed in Figure 2A, 2B, respectively. Functional annotation from Metascape revealed that these differentially-expressed RBPs were involved in multiple critical cellular processes, including ncRNA metabolic process (GO term, *P* < 0.001), mRNA metabolic process (GO term, *P* < 0.001), nucleic acid phosphodiester bond hydrolysis (GO term, *P* < 0.001), mRNA surveillance pathway (KEGG term, *P* < 0.001), regulation of mRNA metabolic process (GO term, *P* < 0.001), metabolism of RNA (Reactome term, *P* < 0.001), DNA methylation or demethylation (GO term, *P* < 0.001), antiviral mechanism by IFN-stimulated genes (Reactome term, *P* < 0.001), negative regulation of viral process (GO term, *P* < 0.001), cellular response to oxidative stress (GO term, *P* < 0.001), mRNA processing (WikiPathways term, *P* < 0.001), RNA 3′-end processing (GO term, *P* < 0.01), diseases of programmed cell death (Reactome term, *P* < 0.01), blastocyst development (GO term, *P* < 0.01), cellular response to DNA damage stimulus (GO term, *P* < 0.01), skeletal system development (GO term, *P* < 0.01), protein autophosphorylation (GO term, *P* < 0.01), and translation (GO term, *P* < 0.01), uncovering the underlying biological functions of these RBPs in the pathogenesis of OA (Figure 2C).

### PPI network analyses

**Figure 3:**
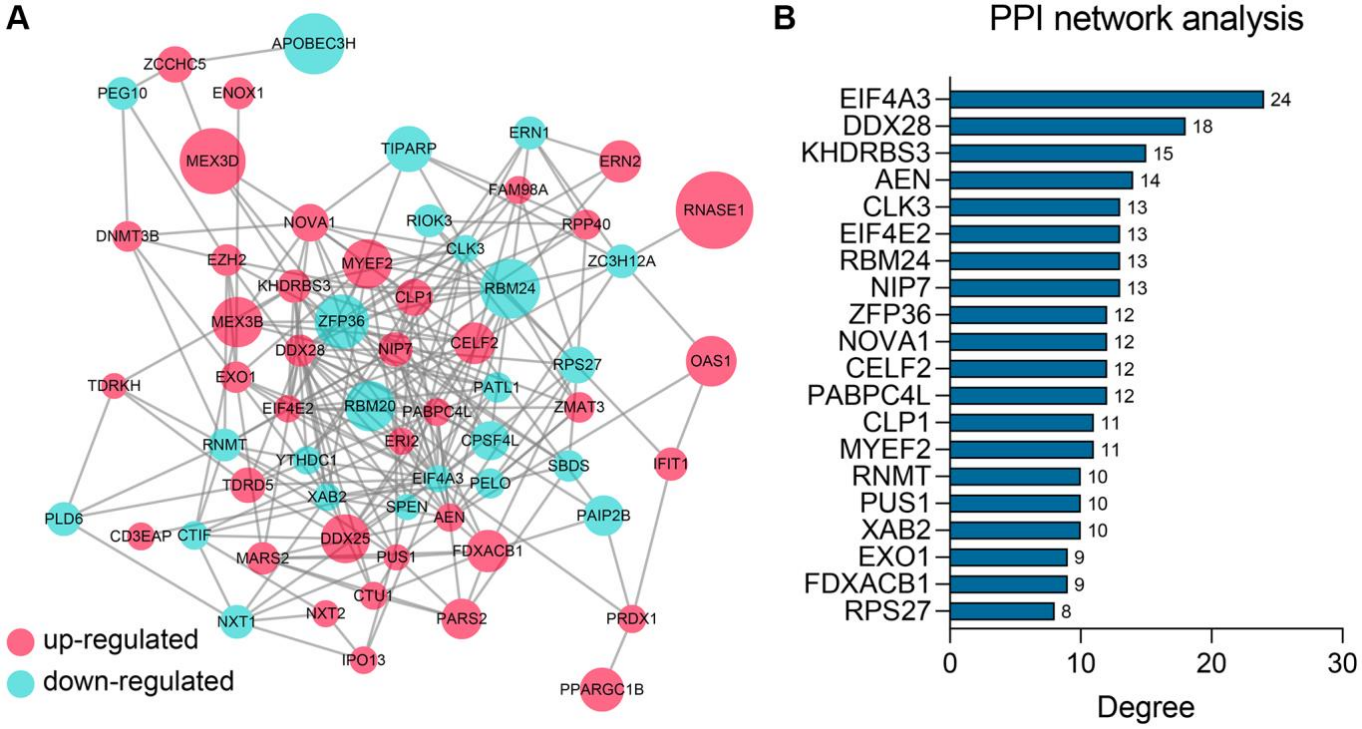
**PPI network construction and analysis.** (**A**) The PPI network of the 62 differentially-expressed RBPs. (**B**) The Top 20 genes ranked by degree in the network. Abbreviation: PPI: protein-protein interaction.

To detect the potential interactions of the 62 genes, we uploaded these genes into the STRING database to construct the PPI network (Figure 3A). Next, the importance of these genes in the network was quantified using the cytoHubba app in Cytoscape (Supplementary Table 2). The Top 20 genes showing the highest degree were chosen for further study, including EIF4A3, DDX28, KHDRBS3, AEN, CLK3, EIF4E2, RBM24, NIP7, ZFP36, NOVA1, CELF2, PABPC4L, CLP1, MYEF2, RNMT, PUS1, XAB2, EXO1, FDXACB, and RPS27 (Figure 3B).

### RBM24 and RNMT were identified as potential diagnostic biomarkers to OA

**Figure 4:**
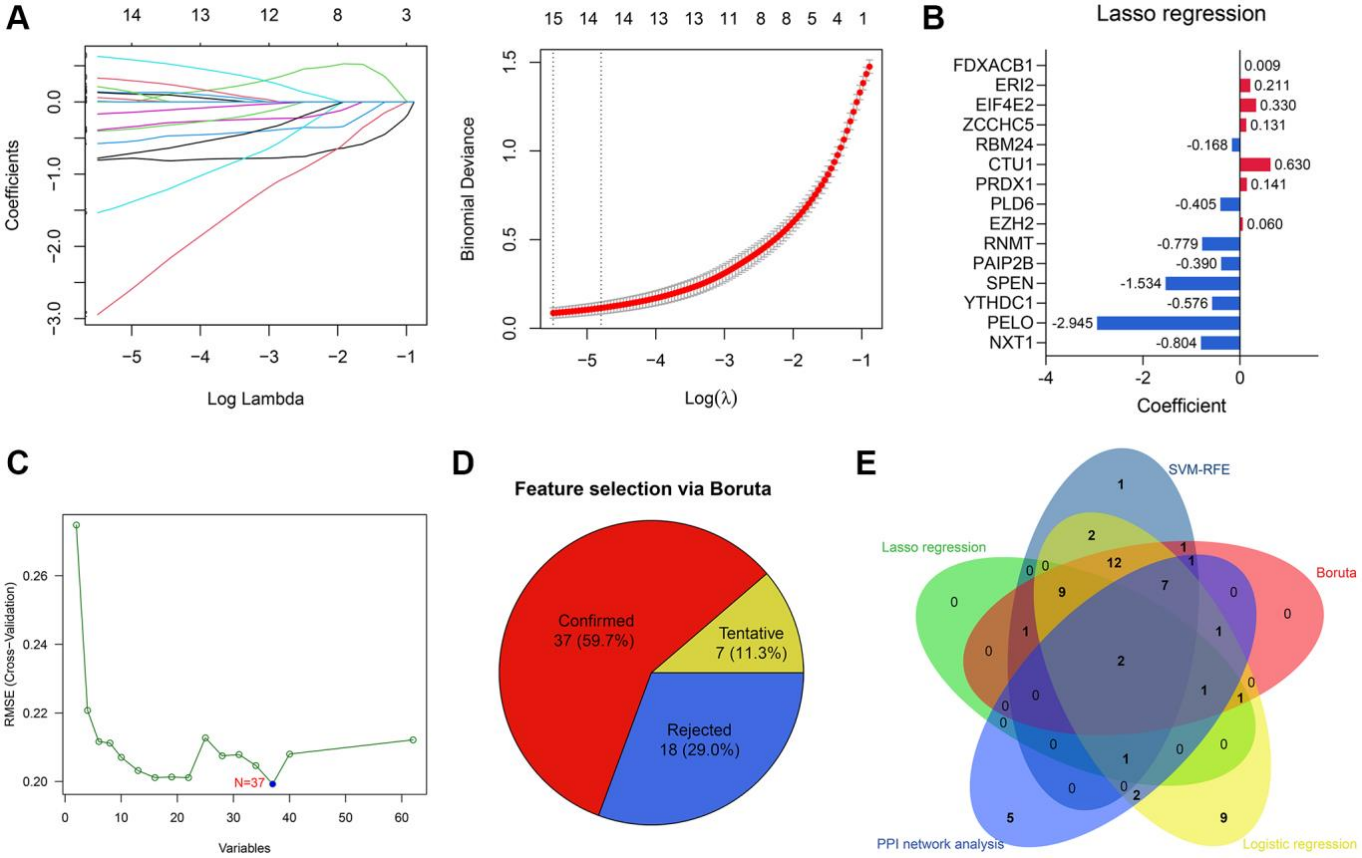
**Feature selection via machine learning algorithms.** (**A**) 15 genes were determined by Lasso regression. (**B**) The parameters of the variables in the Lasso regression model. (**C**) 37 genes were identified by SVM-RFE algorithm. (**D**) Boruta algorithm helped to select 37 genes. (**E**) RNMT and RBM24 were con-determined by the PPI network analysis and machine learning algorithms.

15 of 62 RBPs were determined as significant features to evaluate the possibility of OA development via Lasso regression (Figure 4A). The parameters of these 15 features in the Lasso regression model are shown in Figure 4B. To render the predictive model more concise, we implemented other feature selection methods at the same time. SVM-RFE algorithm identified 37 variables significantly associated with the outcomes (Figure 4C). 47 genes were determined via univariate logistic regression with *P* < 0.01 filtering (Supplementary Table 3), and the Boruta algorithm helped to identify 37 genes (Figure 4D). Ultimately, RNA Guanine-7 Methyltransferase (RNMT) and RNA Binding Motif Protein 24 (RBM24) were con-determined by the feature selection algorithms and PPI network analysis (Figure 4E) and then included in the diagnosis model. To clarify the specificity and sensitivity of RNMT and RBM24, we then performed the ROC analyses in the training cohort. The optimal cut-off value for RBM24 was 0.500, where its specificity and sensitivity were 0.944 and 0.800, respectively (Supplementary Figure 1A). The best cut-off for RNMT was 2.917, and its specificity and sensitivity were 0.778 and 0.900, respectively (Supplementary Figure 1B).

### Construction and external validation of the RF model

**Figure 5:**
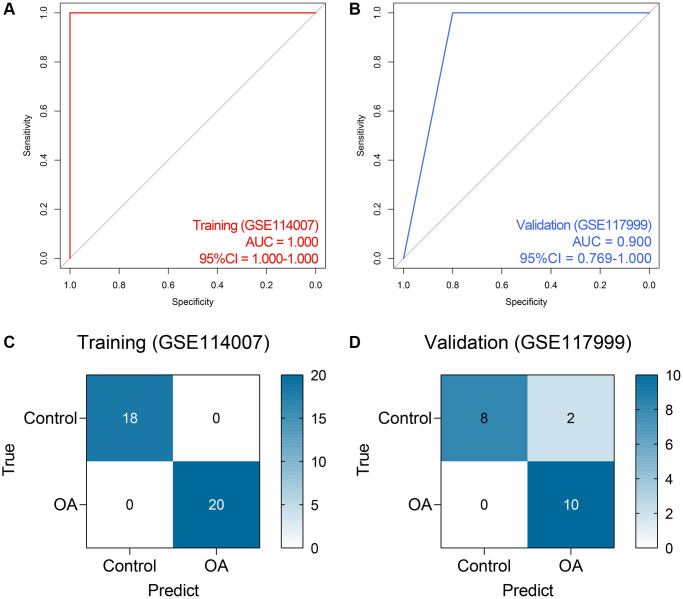
**The performance of the random forest model in the public datasets.** (**A**, **B**) The ROC analysis of the diagnosis model in the training (**A**) and external validation (**B**) datasets. (**C**, **D**) The confusion matrices of the model in the training (**C**) and external validation (**D**) datasets. Abbreviations: ROC: receiver operating characteristic.

Based on the expressions of RBM24 and RNMT, an RF diagnosis model was developed, and the modeling parameters are stated above. The RF model exhibited high efficacy to distinguish the PA samples from the control cases, both in the training dataset (AUC = 1.000, 95% CI = 1.000–1.000, Figure 5A) and the GSE117999 dataset (AUC = 0.900, 95% CI = 0.769–1.000, Figure 5B). The confusion matrices in the training dataset and the GSE117999 dataset were displayed in Figure 5C and Figure 5D, respectively.

**Figure 6:**
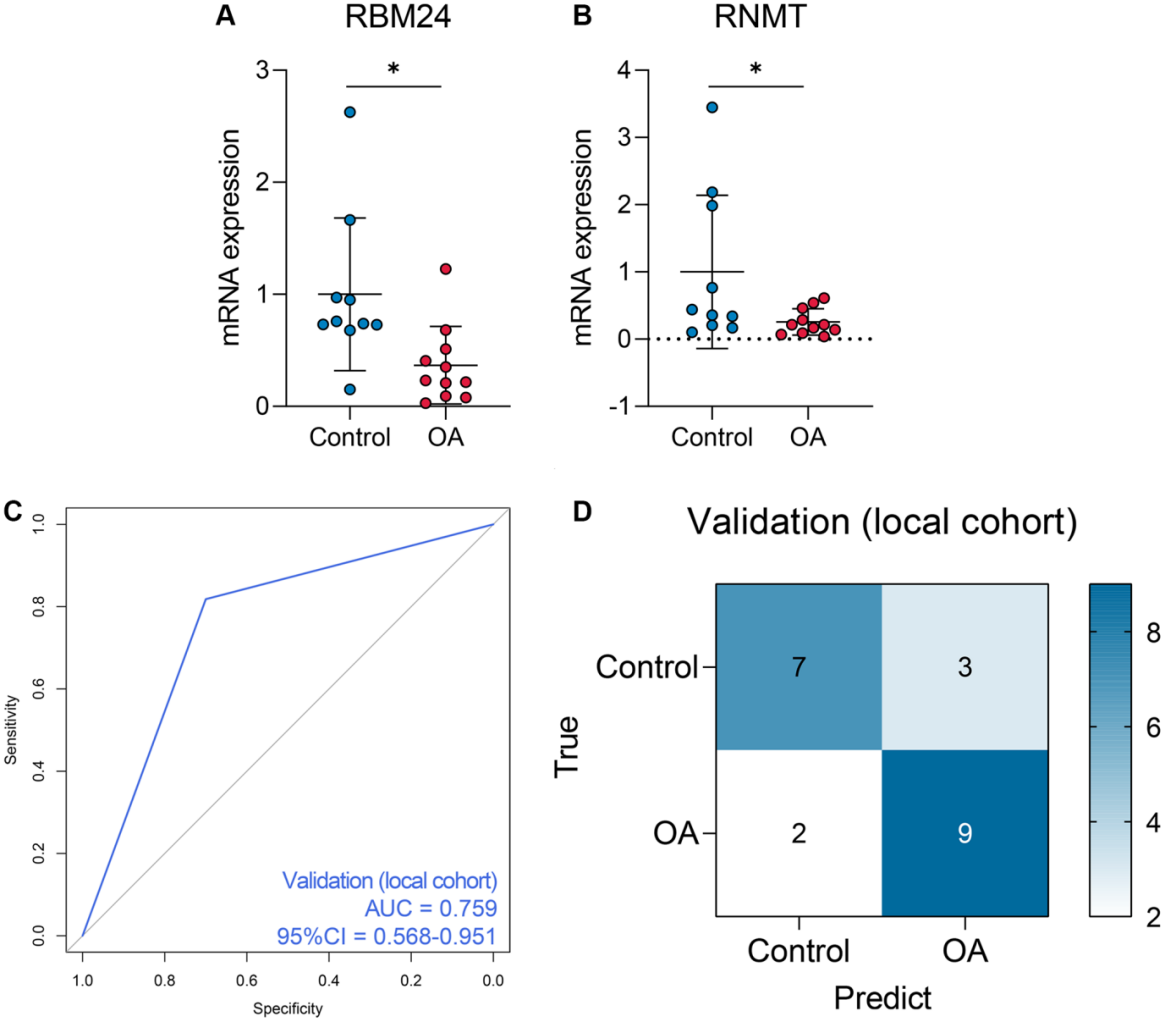
**The performance of the random forest model in the local cohort.** (**A**, **B**) The qPCR experiments indicated that RNMT and RBM24 were both down-regulated in the knee cartilage tissue extracted from OA patients. (**C**, **D**) The ROC analysis (**C**) and the confusion matrix (**D**) of the random forest model in the local cohort.

Given the fact that the GSE117999 dataset has not been publicly published, we collected 10 control and 11 OA samples from the local hospital to re-confirm the reliability of the model. The baseline clinicopathological parameters of the training cohort and the local cohort were displayed in Table 3. Since the GSE117999 dataset has not been published, the clinical information of the GSE117999 cohort was unavailable. The gene expression levels of RBM24 and RNMT were measured via RT-qPCR since the predictive model was based on the RNA expression values. The raw CT values of the genes in the clinical samples were exhibited in Supplementary Table 4. Compared with the control samples, RBM24 (*P* < 0.05, Figure 6A) and RNMT (*P* < 0.05, Figure 6B) were both down-regulated in the OA samples. The RF model could also diagnose OA in the local cohort to some extent (AUC = 0.759, 95% CI = 0.568–0.951, Figure 6C, 6D showed the corresponding confusion matrix. The RF model exhibited moderate diagnosis performance in the local cohort compared with that in the public datasets, and the different gene expression detection platforms (RT-qPCR vs. high-throughput sequencing) and the relatively small sample size might account for this divergence. Other assessment indexes, including accuracy, precision, recall, F-measure, sensitivity, specificity, positive predictive value, and negative predictive value, of the RF model in each cohort were shown in Table 4.

**Table 3:** The baseline clinical traits of the training cohort and the local cohort.

**Parameters**	**GSE114007**		**Local cohort**	
	**Control (*n* = 18)**	**OA (*n* = 20)**	**Control (*n* = 10)**	**OA (*n* = 11)**
Age	–	–	56 ± 8.2	62 ± 7.4
Gender				
Male	13 (72.2%)	8 (40.0%)	4 (40.0%)	7 (63.6%)
Female	5 (27.8%)	12 (60.0%)	6 (60.0%)	4 (36.4%)
BMI	32.4 ± 8.0	30.7 ± 8.1	29.6 ± 9.1	33.5 ± 7.4
Kellgren-Lawrence Grade				
I	–	–	–	0 (0.0%)
II	–	–	–	0 (0.0%)
III	–	–	–	2 (18.2%)
IV	–	–	–	9 (81.8%)
Varus Deformity	–	–	–	10 (90.9%)
Valgus Deformity	–	–	–	1 (9.1%)

Abbreviation: BMI: body mass index. The data is presented as *n* (%) or mean ± standard deviation.

**Table 4:** The predictive performance of the RF model in each cohort.

**Cohort**	**Accuracy**	**Precision**	**Recall**	**F-measure**	**Sensitivity**	**Specificity**	**Positive predictive value**	**Negative predictive value**
GSE114007	1.000	1.000	1.000	1.000	1.000	1.000	1.000	1.000
GSE117999	0.900	0.833	1.000	0.909	1.000	0.800	0.833	1.000
Local Cohort	0.762	0.750	0.818	0.783	0.818	0.700	0.750	0.778

### RBM24 and RNMT were associated with genesis of OA

**Figure 7:**
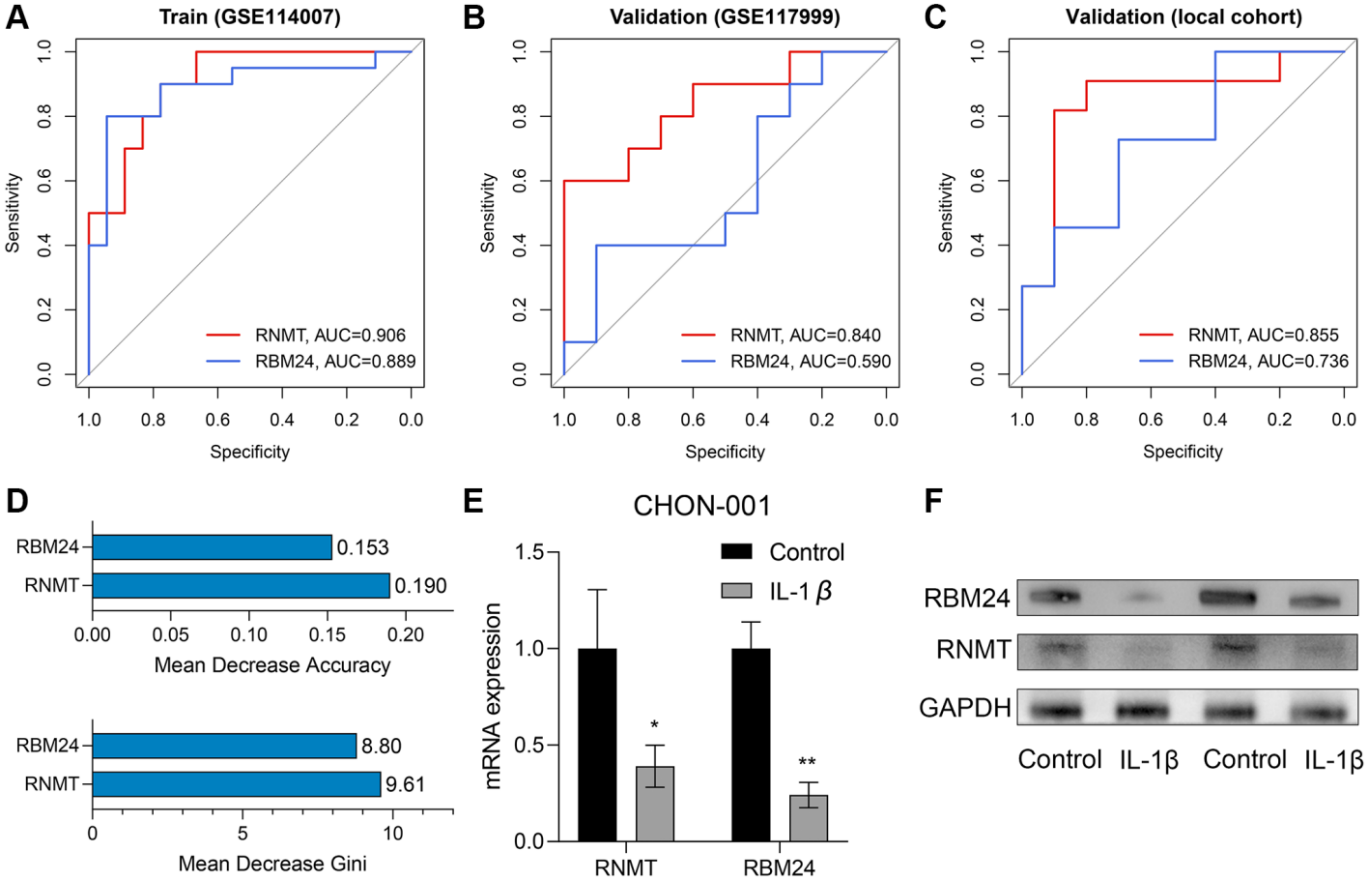
**RNMT and RBM24 were associated with genesis of OA.** (**A**–**C**) The diagnosis value of RNMT and RBM24 in the training cohort (**A**), the GSE117999 cohort (**B**), and the local cohort (**C**). (**D**) The mean decrease accuracy (up) and the mean decrease Gini (bottom) of RNMT and RBM24 in the random forest model. (**E**, **F**) The qPCR experiments (**E**) and Western Blotting (**F**) displayed that RNMT and RBM24 were both down-regulated in the CHON-001 cells treated with 10 ng/mL IL-1β.

The ROC analyses revealed the predictive ability of RBM24 and RNMT to OA in the training dataset (RNMT, AUC = 0.906; RBM24, AUC = 0.889; Figure 7A), the GSE117999 dataset (RNMT, AUC = 0.840; RBM24, AUC = 0.590; Figure 7B), and the local cohort (RNMT, AUC = 0.855; RBM24, AUC = 0.736; Figure 7C). We observed that except for RBM24 in the GSE117999 cohort, the variables in these cohorts all exhibited high diagnosis values (AUC > 0.7). At the same time, the mean decrease accuracy and mean decrease Gini of RNMT were superior to those of RBM24 (Figure 7D), suggesting that RNMT was a relatively more reliable biomarker than RBM24. At last, we constructed the OA model *in vitro* and detected the RNA and protein levels of RNMT and RBM24. RT-qPCR and Western Blotting indicated that RNMT (*P* < 0.05) and RBM24 (*P* < 0.01) were both down-regulated in the OA model group, as shown in Figure 7E, 7F respectively. This evidence demonstrated that RBM24 and RNMT both serve as critical biomarkers for OA and are associated with OA development.

### The functional annotation of RBM24 and RNMT

**Figure 8:**
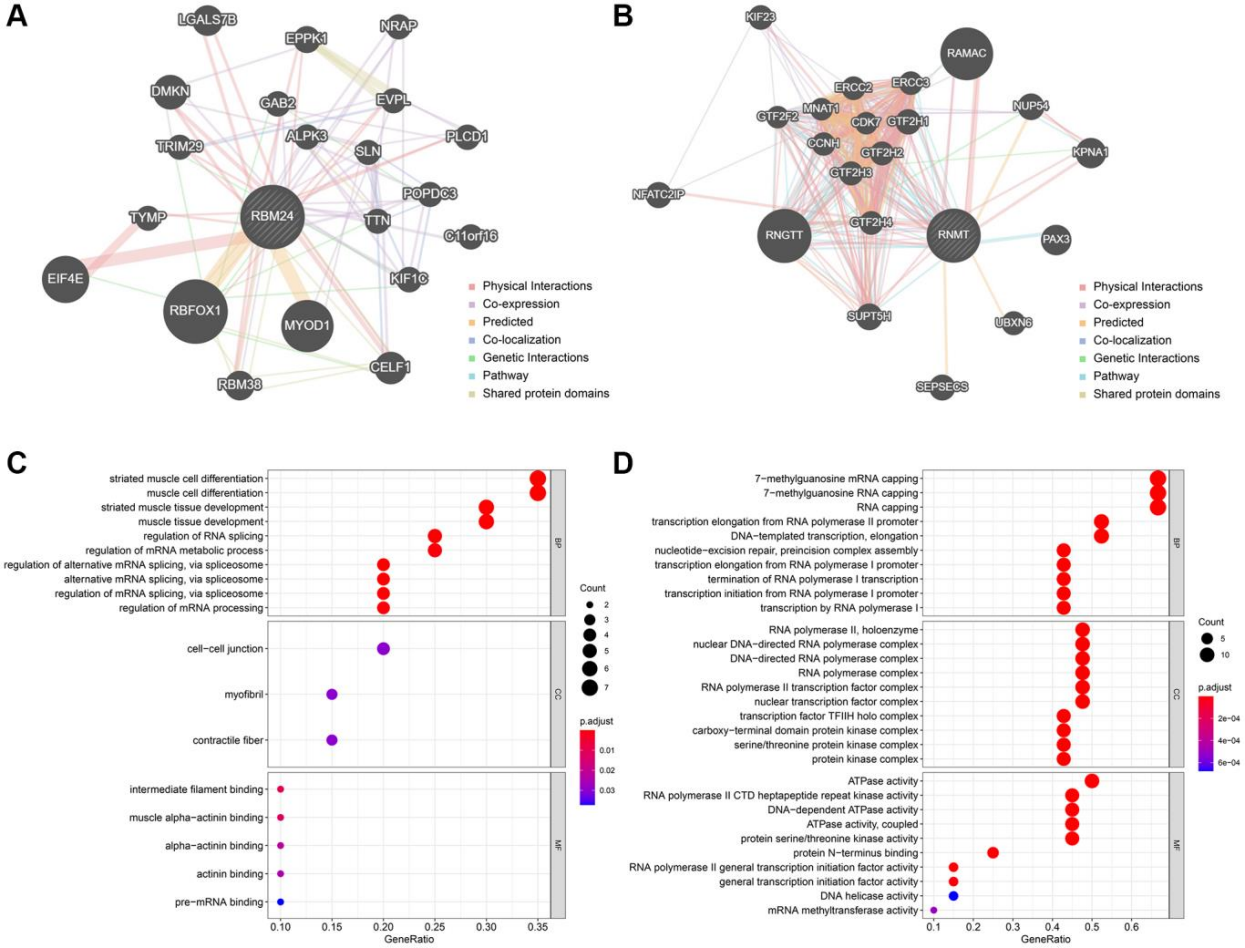
**The functionally-associated genes and GO enrichment.** (**A**, **B**) The Top 20 genes associated with RBM24 (**A**) and RNMT (**B**). GO functional annotation of the associated genes of RBM24 (**C**) and RNMT (**D**). Abbreviation: GO: gene ontology.

The Top 20 genes most relevant to RBM24 and RNMT were illustrated in Figure 8A and Figure 8B, respectively. Gene Ontology (GO) analysis indicated that RBM24 and its associated genes were mainly enriched in muscle development and differentiation and RNA metabolism, splicing, and processing (Figure 8C), and RNMT and its correlated genes mainly participated in RNA synthesis and modification, ATPase activity, and serine/threonine kinase activity (Figure 8D). These results might provide clues to elucidate the roles RBM24 and RNMT play in OA.

## DISCUSSION

Currently, the diagnosis of OA mainly depends on the clinical presentations and imaging examination, often leading to delayed diagnosis and missing the optimal time to intervene [21]. Therefore, many efforts have been devoted to seeking reliable diagnosis biomarkers in recent years, and some novel molecules as OA diagnosis markers have been reported, such as ATF3 [22], Apolipoprotein D [23], and CXCL13 [24]. The rapid development of genomic sequencing technology and big-data analysis methods represented by machine learning generates new opportunities to disclose novel biomarkers and to develop novel diagnostic tools, and many achievements have been obtained in OA [25–27]. Nevertheless, the reports about OA diagnosis models based on machine algorithms are relatively rare at the moment.

As important posttranscriptional regulators, RBPs are capable of modulating RNA metabolism and thus affect the expression levels of proteins [28]. The huge effects of RBPs on gene expression enlighten the investigators to study the roles RBPs play in OA, and some RBPs, such as GNL3 [29], SND1 [30], and ZFP36L1 [31], have been verified as critical regulators in the pathogenesis of OA. However, the number of RBP-related research in OA is still limited for the moment.

Herein, the present study collected 1525 RBPs from previous reports and detected their expression levels in the knee cartilage tissue isolated from control and OA subjects. A sum of 62 differentially-expressed RBPs were identified, RNMT and RBM24 of which were determined as the significant biomarkers for OA through multiple machine learning algorithms and bioinformatical analyses. To achieve better predictive performance, we constructed an RF model using the expression values of RNMT and RBM24. Importantly, the RF model has been externally validated in another public dataset and the clinical samples collected from the local hospital. Besides, the *in vitro* OA model was constructed in CHON-001 cells, which were treated with IL-1β to mimic OA. The RNA and protein levels of RNMT and RBM24 significantly decreased in the OA model group, suggesting RNMT and RBM24 might be important regulators in the development of OA.

We firstly reported that RNMT and RBM24 acted as potential biomarkers for OA and conducted the preliminary verification in the OA cell model and clinical samples. RNMT, a regulatory factor for 7-methylguanosine mRNA capping, exerts inhibitory effects against 5′-exonucleases and promotes RNAs’ export and translation [32]. It is worth mentioning that the polarity of RNMT ligands is low, rendering the compounds targeting this molecule easier to cross the plasma membrane [33]. Alison et al., reported that RNMT was an essential mediator for T cell activation [34]; meanwhile, many studies have demonstrated that T cells exhibit high numbers in OA samples compared with the control samples [35], but their correlations need to be further clarified. RBM24, a highly conserved RBP, is an important regulator for cell differentiation and cellular homeostasis [36]. RBM24 was reported to be recruited in stress granules, which were formed in cells under stress, and to protect its target RNAs [37], which might be the underlying biological function of RBM24 in OA development. However, more direct experimental evidence supporting these assumptions in OA is demanded in future studies.

The shortcomings of this study should be stated. First, although the RF model was validated in the clinical samples from the local hospital, a prospective, large-scale, and multi-center clinical trial would be helpful to better clarify its usefulness. Additionally, the roles of RNMT, RBM24, and the RF model in OA’s early detection or prognosis prediction are unknown, which is the research direction in the future. Second, more experimental exploration should be performed to elucidate the biological functions and underlying mechanisms of RNMT and RBM24 in OA. Third, although CHON-001 cells treated with IL-1β were widely used for *in vitro* OA model construction, it should be emphasized that CHON-001 cells were isolated from the cartilage tissue of an embryo (age 18 weeks) and exhibited a fibroblast-like morphology, leading to different characteristics compared with primary chondrocytes. For instance, adult chondrocytes rarely divide throughout life, but CHON-001 cells are proliferative cells. This is the limitation of the methodology in this study.

In conclusion, an RF model based on RNMT and RBM24 was established to diagnose OA, which was externally validated in public datasets, local clinical samples, and *in vitro* cell experiments.

## Supplementary Materials

Supplementary File 1

Supplementary Figure 1

Supplementary Table 1

Supplementary Tables 2-4
