# Supplementary material for: A two-gene random forest model to diagnose osteoarthritis based on RNA-binding protein-related genes in knee cartilage tissue
Source: Aging (Albany NY). 2023 Jan 5;15(1):193–212. doi: 10.18632/aging.204469 (PMC9876643; doi:10.18632/aging.204469)
Supplement: Supplementary File 1 [file aging-15-204469-s001.pdf]

Supplementary File 1. The R codes used in this study.

```
#Genomic difference analysis
library(limma)
logFoldChange=1
adjustP=0.05
rt=read.table("input.txt",sep="\t",header=T,check.names=F)
rt=as.matrix(rt)
rownames(rt)=rt[,1]
exp=rt[,2:ncol(rt)]
dimnames=list(rownames(exp),colnames(exp))
rt=matrix(as.numeric(as.matrix(exp)),nrow=nrow(exp),dimnames=dimna
mes)
rt=avereps(rt)
modType=c(rep("con",conNum),rep("treat",treatNum))
design <- model.matrix(~0+factor(modType))
colnames(design) <- c("con","treat")
fit <- lmFit(rt,design)
cont.matrix<-makeContrasts(treat-con,levels=design)
fit2 <- contrasts.fit(fit, cont.matrix)
fit2 <- eBayes(fit2)
allDiff=topTable(fit2,adjust='fdr',number=200000)
write.table(allDiff,file="All_limma.xls",sep="\t",quote=F)
```

```
#The volcano plot
library(ggpubr)
library(ggthemes)
deg.data <- read.table("score.cor.txt", header = T, sep = "\t")
head(deg.data)
deg.data$logp <- -log10(deg.data$FDR)
deg.data$group = "notsignificant"
deg.data$group[which((deg.data$FDR < 0.05) & (deg.data$logFC > 0))]
= "up"
deg.data$group[which((deg.data$FDR < 0.05) & (deg.data$logFC < 0))]
= "down"
table(deg.data$group)
deg.data$label = ""
deg.data <- deg.data[order(deg.data$FDR),]
up.genes <- head(deg.data$gene[which(deg.data$group == "up")], 0)
down.genes <- head(deg.data$gene[which(deg.data$group == "down")],
0)
deg.top10.genes <- c(as.character(up.genes),as.character(down.genes))
deg.data$label[match(deg.top10.genes, deg.data$gene)] <-
deg.top10.genes
pdf(file="volcano.pdf",
    width = 5,
```

```
height = 4,
)
ggscatter(deg.data, x = "logFC", y = "logp", color = "group", palette = c("#4169E1","#BBBBBB","#E3170D"), size = 1, label = deg.data$label, font.label = 8, repel = T, xlab = "Spearman Correlation", ylab = "-log10P",) + theme_base() + geom_hline(yintercept = 1.30, linetype = "dashed") + geom_vline(xintercept = c(0,0), linetype = "dashed")
dev.off()


#GO functional enrichment
library("org.Hs.eg.db")
rt=read.table("DEGs.txt",sep="\t",check.names=F,header=T)
genes=as.vector(rt[,1])
entrezIDs <- mget(genes, org.Hs.egSYMBOL2EG, ifnotfound=NA)
entrezIDs <- as.character(entrezIDs)
out=cbind(rt,entrezID=entrezIDs)
write.table(out,file="id.txt",sep="\t",quote=F,row.names=F)
library("clusterProfiler")
library("enrichplot")
library("ggplot2")
rt=read.table("id.txt",sep="\t",header=T,check.names=F)
```

```
rt=rt[is.na(rt[,"entrezID"])==F,]
gene=rt$entrezID
kk <- enrichGO(gene = gene,
               OrgDb = org.Hs.eg.db,
               pvalueCutoff =0.05,
               qvalueCutoff = 0.05,
               ont="all",
               readable =T)
write.table(kk,file="GO.txt",sep="\t",quote=F,row.names = F)

pdf(file="bubble_go.pdf",width = 10,height = 8)
dotplot(kk,showCategory = 10,split="ONTOLOGY") + facet_grid(ONTOLOGY~., scale='free')
dev.off()

#Lasso
library(glmnet)
set.seed(1234)
mydata<-read.table("test.txt",header=T,sep="\t",row.names = 1)
v1<-as.matrix(mydata[,c(2:ncol(mydata))])
v2 <-mydata[,1]
myfit <- glmnet(v1, v2, family = "binomial",alpha=1)
```

```
pdf("lambda.pdf")
plot(myfit, xvar = "lambda", label = TRUE)
dev.off()
myfit2 <- cv.glmnet(v1, v2, family="binomial",alpha=1,type.measure='auc',nfolds = 10)
pdf("min.pdf")
plot(myfit2)
dev.off()
myfit2$lambda.min
coef <- coef(myfit, s = myfit2$lambda.min)
index=which(coef != 0)
actCoef=coef[index]
lassoGene=row.names(coef)[index]
coef=cbind(Gene=lassoGene,Coef=actCoef)
write.table(coef,file="LASSO_coef.txt",sep="\t",quote=F,row.names=F)
lassoGene=lassoGene[which(lassoGene !="(Intercept)")]
lassoGene=c("status",lassoGene)
lassoGeneSigExp=mydata[,lassoGene]
lassoGeneSigExp=cbind(id=row.names(mydata),lassoGeneSigExp)
write.table(lassoGeneSigExp,file="lassoGeneSigExp.txt",sep="\t",row.names=F,quote=F)
```

```
#Boruta
train <-
read.table("test.txt",sep="\t",header=T,check.names=F,row.names=1)
str(train)
library(Boruta)
set.seed(123)
feature.selection <- Boruta(status ~ ., data = train, doTrace = 1)
pdf("feature_selection.pdf",width = 20,height = 20)
plot(feature.selection)
dev.off()
feature.selection$timeTaken
feature.selection$finalDecision
final.decision <- feature.selection$finalDecision
class(final.decision)
H<-table(feature.selection$finalDecision)
pdf("barplot.pdf",width = 6,height = 6)
barplot(H,col=c("#FFFF00","#3D9140","#B0171F"),main="Feature
selection with Random Forest")
dev.off()
pdf("pie.pdf",width = 6,height = 6)
pie(H,col=c("#FFFF00","#3D9140","#B0171F"),main="Feature selection
with Random Forest")
```

```
dev.off()
mydata <- data.frame(H)
library(ggplot2)
library(RColorBrewer)
pdf("barplot_ggplot2.pdf",width = 4,height = 4)
ggplot(data=mydata,aes(Var1,Freq))+
  geom_bar(stat="identity", color="black", width=0.6,fill="#FC4E07",size=0.25) +#"#00AFBB"
  scale_fill_manual(values=brewer.pal(9,"YlOrRd")[c(6:2)])+
  labs(x = "", y = "", title = "Feature selection with Random Forest")+
  coord_flip()+
  theme(
    axis.title=element_blank(),
    axis.text = element_text(size=12,face="plain",color="black"),
    legend.title=element_text(size=13,face="plain",color="black"),
    legend.position = "right"# c(0.83,0.15)
  )
dev.off()

fNames <- getSelectedAttributes(feature.selection)
fNames
randomForestGene= gsub(pattern = "`", replacement = "", x = fNames)
```

```
randomForestGene=c("status",randomForestGene)
randomForestGeneSigExp=train[,randomForestGene]
randomForestGeneSigExp=cbind(id=row.names(randomForestGeneSigE
xp),randomForestGeneSigExp)
write.table(randomForestGeneSigExp,file="randomForestGeneSigExp.txt
",sep="\t",row.names=F,quote=F)

#SVM
library(e1071)
library(kernlab)
library(caret)


set.seed(123)
inputFile="diffGeneExp.txt"
data=read.table(inputFile,    header=T,    sep="\t",    check.names=F,
row.names=1)
data=t(data)
group=gsub("(.*)\\_(.*)", "\\2", row.names(data))

Profile=rfe(x=data,
            y=as.numeric(as.factor(group)),
            sizes = c(2,4,6,8, seq(10,40,by=3)),
```

```
            rfeControl = rfeControl(functions = caretFuncs, method =
"cv"),
            methods="svmRadial")

pdf(file="SVM-RFE.pdf", width=6, height=5.5)
par(las=1)
x = Profile$results$Variables
y = Profile$results$RMSE
plot(x, y, xlab="Variables", ylab="RMSE (Cross-Validation)",
col="darkgreen")
lines(x, y, col="darkgreen")
wmin=which.min(y)
wmin.x=x[wmin]
wmin.y=y[wmin]
points(wmin.x, wmin.y, col="blue", pch=16)
text(wmin.x, wmin.y, paste0('N=',wmin.x), pos=2, col=2)
dev.off()
featureGenes=Profile$optVariables
write.table(file="SVM-RFE.gene.txt", featureGenes, sep="\t", quote=F,
row.names=F, col.names=F)

#univariate logistic regression
```

```
library(rms)
library(pROC)
rt<-read.table("pre_RF.txt",header=T,sep="\t",check.names=F,row.names
=1)
ddist <- datadist(rt)
options(datadist="ddist")
pFilter <- 0.05
sigGenes=c("status")
outTab=data.frame()
for(i in colnames(rt[,2:ncol(rt)])){
  mymodel <- glm(status~rt[,i],family=binomial(link = "logit"),data = rt)
  mymodelSummary = summary(mymodel)
  mymodelP=mymodelSummary$coefficients[2,"Pr(>|z|)"]
  predict <- predict.glm(mymodel,type = "response",newdata = rt)
  true_value = rt[,1]
  modelroc <- roc(true_value,predict)
  AUC = modelroc$auc
  outTab=rbind(outTab,
               cbind(id=i,
                     OR=matrix(exp(coefficients(mymodel)))[2,1],
                     OR.95L=exp(confint(mymodel))[2,"2.5 %"],
                     OR.95H=exp(confint(mymodel))[2,"97.5 %"],
```

```
pvalue=mymodelSummary$coefficients[2,"Pr(>|z|)"],
                                AUC=AUC)
   )
   if(mymodelP<pFilter){
      sigGenes=c(sigGenes,i)
   }
}
write.table(outTab,file="unilogit.xls",sep="\t",row.names=F,quote=F)
uniSigExp=rt[,sigGenes]
uniSigExp=cbind(id=row.names(uniSigExp),uniSigExp)
write.table(uniSigExp,file="uniSigExp.txt",sep="\t",row.names=F,quote=
F)

#Random forest model construction and ROC analysis
library(ROCit)
library(randomForest)
library(rms)
library(pROC)
train_data = read.table("train.txt",header=T,sep="\t",check.names =
F,row.names = 1)
test_data = read.table("test.txt",header=T,sep="\t",check.names =
```

```
F,row.names = 1)
train_data$Group = as.factor(train_data$Group)
test_data$Group = as.factor(test_data$Group)
train_data$Group = as.factor(train_data$Group)
test_data$Group = as.factor(test_data$Group)
train_randomforest <- randomForest(Group ~.,
                                   data = train_data,
                                   ntree =500,
                                   mtry=3,
                                   importance=TRUE ,
                                   proximity=TRUE)
train_randomforest$importance
varImpPlot(train_randomforest, main = "variable importance")
pdf(file="train_randomforest_importance.pdf",
    width = 5,
    height = 4,
)
varImpPlot(train_randomforest, main = "variable importance")
dev.off()

#Train ROC
library(pROC)
```

```
pre_ran <- predict(train_randomforest,newdata=train_data)
train_data$pre_ran <- pre_ran
train_data$pre_ran <- as.numeric(train_data$pre_ran)
obs_p_ran = data.frame(prob=pre_ran,obs=train_data$Group)
table(train_data$Group,pre_ran,dnn=c("True value","Predicted value"))
ran_roc <- roc(train_data$Group,as.numeric(pre_ran))
plot(ran_roc, print.auc=TRUE, auc.polygon=TRUE, grid=c(0.1, 0.2),grid.col=c("green", "red"), main='Training dataset (GSE66360)')
pdf("ROC_train_randomforest.pdf",6,6)
plot(ran_roc, print.auc=TRUE, col="#4169E1")
dev.off()

#Test ROC
pre_ran <- predict(train_randomforest,newdata=test_data)
test_data$pre_ran <- pre_ran
test_data$pre_ran <- as.numeric(test_data$pre_ran)
obs_p_ran = data.frame(prob=pre_ran,obs=test_data$Group)
table(test_data$Group,pre_ran,dnn=c("True value","Predicted value"))
ran_roc <- roc(test_data$Group,as.numeric(pre_ran))
plot(ran_roc, print.auc=TRUE, auc.polygon=TRUE, grid=c(0.1, 0.2),grid.col=c("green", "red"), main='Training dataset (GSE66360)')
pdf("ROC_test_randomforest.pdf",6,6)
```

```
plot(ran_roc, print.auc=TRUE, col="#4169E1")
dev.off()


#The calculation of the areas under the receiver operating curve.
library(pROC)
inputFile="input.txt"
outFile="ROC.pdf"
rt=read.table(inputFile,header=T,sep="\t",check.names=F,row.names=1)
y=colnames(rt)[1]
bioCol=c("#E3170D","#4169E1","#03A89E","#03A89E")
if(ncol(rt)>4){
    bioCol=rainbow(ncol(rt))}


pdf(file=outFile,width=4.5,height=4.5)
roc1=roc(rt[,y], as.vector(rt[,2]))
aucText=c( paste0(colnames(rt)[2],", AUC=",sprintf("%0.3f",auc(roc1))) )
plot(roc1, col=bioCol[1])
for(i in 3:ncol(rt)){
    roc1=roc(rt[,y], as.vector(rt[,i]))
    lines(roc1, col=bioCol[i-1])
    aucText=c(aucText, paste0(colnames(rt)[i],",
```

```
AUC=",sprintf("%0.3f",auc(roc1))) )
}
legend("bottomright", aucText,lwd=2,bty="n",col=bioCol[1:(ncol(rt)-1)])
dev.off()
```
