# Supplementary material for: A two-gene random forest model to diagnose osteoarthritis based on RNA-binding protein-related genes in knee cartilage tissue
Source: Aging (Albany NY). 2023 Jan 5;15(1):193–212. doi: 10.18632/aging.204469 (PMC9876643; doi:10.18632/aging.204469)
Supplement: Supplementary Figure 1 [file aging-15-204469-s002.pdf]

SUPPLEMENTARY FIGURE

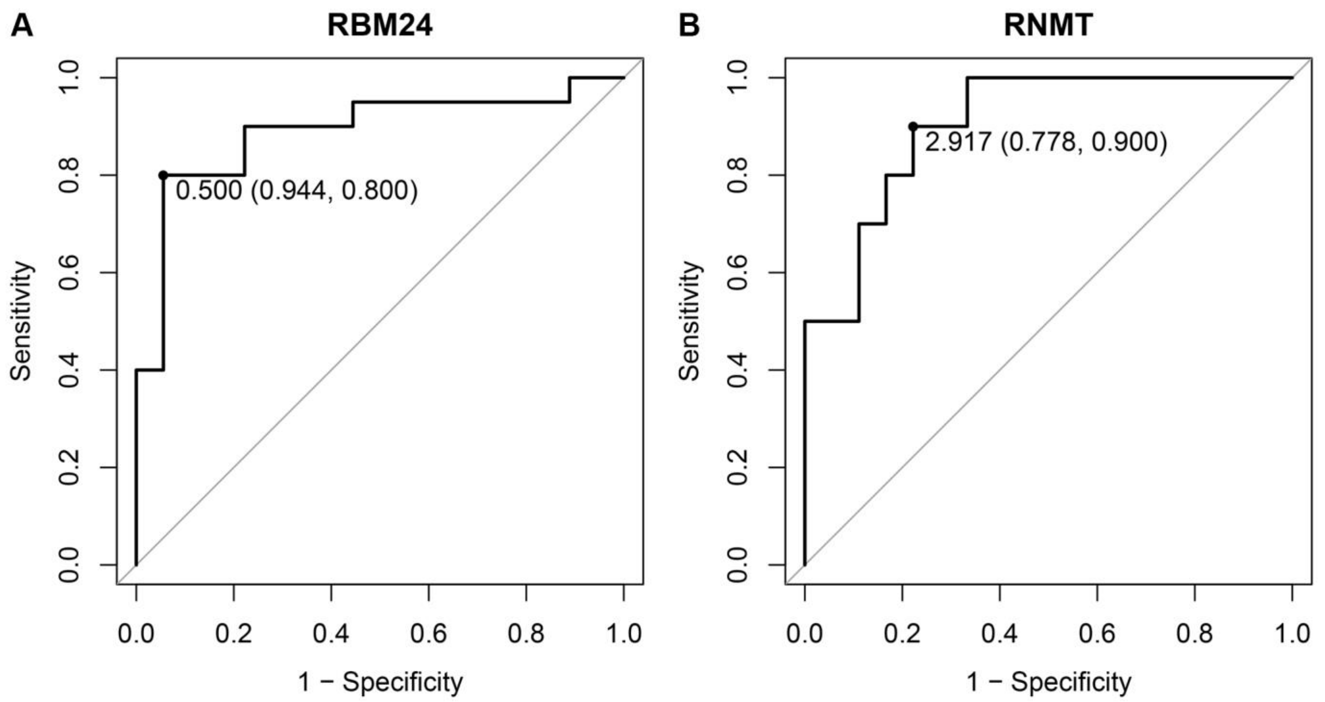

Supplementary Figure 1. The optimal cut-off values and their corresponding specificity and sensitivity of RBM24 (**A**) and RNMT (**B**).
