# Supplementary material for: A two-gene random forest model to diagnose osteoarthritis based on RNA-binding protein-related genes in knee cartilage tissue
Source: Aging (Albany NY). 2023 Jan 5;15(1):193–212. doi: 10.18632/aging.204469 (PMC9876643; doi:10.18632/aging.204469)
Supplement: Supplementary Tables 2-4 [file aging-15-204469-s004.pdf]

SUPPLEMENTARY TABLES

Supplementary Table 2. The importance of the genes in the PPI network.

node_name	MCC	DMNC	MNC	Degree	EPC	Bottle Neck	Ec Centricity	Closeness	Radiality	Betweenness	Stress	Clustering Coefficient
RNASE1	1	0	1	1	4.847	1	0.2	19.71667	3.77049	0	0	0
PPARGC1B	1	0	1	1	3.284	1	0.16667	18.68333	3.60656	0	0	0
PLD6	5	0.30898	3	4	14.959	1	0.2	24.65	4.29508	8.83413	34	0.33333
RIOK3	7	0.46346	3	4	15.012	1	0.2	24.61667	4.31148	4.0303	10	0.5
SBDS	6	0.30898	3	5	17.239	1	0.25	27.5	4.62295	32.16725	132	0.2
TDRKH	5	0.30898	3	4	15.913	2	0.25	26.08333	4.5082	14.6171	68	0.33333
MEX3D	4	0.30779	2	4	15.241	3	0.25	26.58333	4.57377	188.86849	586	0.16667
ENOX1	1	0	1	1	5.764	1	0.2	20.95	4	0	0	0
RPS27	22	0.29157	8	8	21.191	4	0.2	30.28333	4.78689	57.34252	228	0.35714
IPO13	5	0.30898	3	4	14.444	3	0.2	27.2	4.62295	34.32918	166	0.33333
PRDX1	4	0	1	4	11.589	4	0.2	26.95	4.59016	147.75238	372	0
PEG10	3	0.30779	2	3	8.566	1	0.2	22.11667	4.01639	45.47489	140	0.33333
DNMT3B	7	0.2842	4	5	16.202	2	0.25	27.83333	4.67213	57.32172	260	0.3
PELO	5	0.30779	2	5	18.033	1	0.2	27.7	4.62295	18.74944	84	0.2
FAM98A	12	0.47366	4	4	17.613	1	0.2	27.45	4.63934	0.7	4	0.83333
TDRD5	28	0.45378	5	7	21.877	3	0.2	29.45	4.7377	51.78556	218	0.38095
PATL1	52	0.40246	7	7	24.155	1	0.25	30.75	4.90164	20.30137	132	0.52381
PARS2	26	0.56839	4	6	19.344	1	0.2	26.86667	4.4918	14.7224	54	0.4
CTU1	32	0.42794	6	6	21.549	1	0.2	28.2	4.63934	7.53108	52	0.6
RNMT	12	0.2842	4	10	25.452	4	0.25	32.25	4.93443	165.03031	636	0.11111
NXT1	12	0.32413	5	7	20.419	1	0.2	29.78333	4.77049	80.40835	338	0.2381
NXT2	4	0.30898	3	3	10.828	1	0.16667	22.15	4	1.65476	10	0.66667
CTIF	9	0.2842	4	7	19.976	2	0.2	29.53333	4.7541	80.61883	390	0.14286
RPP40	11	0.32413	5	6	19.265	2	0.2	27.45	4.55738	33.51416	124	0.33333
CPSF4L	19	0.45378	5	6	23.226	1	0.25	30.58333	4.91803	9.97532	78	0.46667
EZH2	10	0.2842	4	8	21.669	2	0.25	30.75	4.86885	155.14999	572	0.14286
CLP1	34	0.31026	9	11	28.098	3	0.25	33.58333	5.04918	119.79573	576	0.23636
ERN1	9	0.25931	5	6	20.189	1	0.2	27.53333	4.55738	31.67548	112	0.26667
TIPARP	7	0.2842	4	5	18.085	2	0.25	28	4.65574	27.03146	102	0.3
SPEN	6	0.30898	3	5	17.382	2	0.25	27.5	4.62295	29.50759	94	0.2
IFIT1	3	0	1	3	9.69	1	0.2	24.36667	4.32787	26.23653	62	0
YTHDC1	10	0.25931	5	7	23.735	1	0.25	31.33333	4.95082	44.47497	218	0.2381
MYEF2	42	0.27149	11	11	27.945	1	0.25	32.91667	4.96721	76.29101	368	0.29091
ERN2	4	0.30898	3	3	14.205	1	0.2	24.95	4.39344	0.58333	4	0.66667
ZFP36	23	0.23866	9	12	27.219	9	0.25	33.66667	5.01639	240.56739	718	0.15152
NOVA1	95	0.3733	11	12	28.321	2	0.33333	34.16667	5.08197	166.62022	676	0.33333
CLK3	31	0.25938	10	13	28.181	2	0.25	34.58333	5.08197	236.89318	858	0.17949
RBM20	50	0.47549	6	6	22.614	1	0.25	29.91667	4.85246	11.90253	72	0.66667
KHDRBS3	149	0.38288	14	15	30.382	2	0.33333	36.33333	5.19672	255.50036	1018	0.32381

MEX3B	13	0.28529	6	7	22.503	1	0.25	29.5	4.77049	39.68178	198	0.28571
DDX25	51	0.47549	6	7	23.388	1	0.2	30.36667	4.83607	18.70413	110	0.47619
EIF4E2	88	0.29378	13	13	30.084	1	0.25	34.91667	5.11475	116.73785	632	0.29487
PAIP2B	4	0	1	4	15.943	1	0.2	26.61667	4.54098	23.74821	88	0
CELF2	54	0.33919	10	12	27.883	4	0.25	33.41667	5	133.31217	520	0.25758
CD3EAP	1	0	1	1	5.89	1	0.2	21.28333	4.04918	0	0	0
ZCCHC5	3	0	1	3	5.698	2	0.2	20.56667	3.77049	122.75362	364	0
APOBEC3H	1	0	1	1	2.304	1	0.16667	15.35	2.78689	0	0	0
ZC3H12A	8	0.30898	3	7	18.743	3	0.25	29.5	4.7541	164.1295	490	0.14286
ERI2	6	0.30779	2	6	21.132	1	0.2	29.86667	4.81967	38.27614	164	0.13333
DDX28	168	0.27914	18	18	31.26	7	0.25	38.08333	5.2623	260.52881	1046	0.24837
EXO1	15	0.28529	6	9	24.219	4	0.25	32.25	4.98361	202.10351	666	0.19444
OAS1	3	0.30779	2	3	11.445	2	0.2	24.95	4.40984	16.05	48	0.33333
PABPC4L	128	0.45891	10	12	28.914	1	0.25	34	5.04918	108.82034	496	0.34848
RBM24	90	0.30655	13	13	29.633	2	0.25	34.75	5.09836	136.56635	586	0.30769
PUS1	59	0.38186	9	10	25.75	3	0.2	32.03333	4.90164	79.96712	330	0.35556
XAB2	28	0.32073	8	10	26.788	4	0.25	33.08333	5.03279	192.58545	738	0.24444
ZMAT3	6	0.30779	2	6	21.248	1	0.25	29.41667	4.78689	48.69737	194	0.13333
MARS2	44	0.43905	7	7	21.926	1	0.2	29.53333	4.7541	26.50462	116	0.57143
EIF4A3	231	0.26149	23	24	32.539	8	0.25	41.75	5.42623	753.02289	2586	0.19565
FDXACB1	49	0.4082	8	9	24.676	1	0.2	31.03333	4.83607	62.82318	266	0.38889
NIP7	68	0.30655	13	13	28.032	2	0.2	34.61667	5.06557	168.88007	710	0.30769
AEN	68	0.32197	12	14	29.865	2	0.2	35.61667	5.13115	276.14754	1026	0.25275

Supplementary Table 3. The univariate logistic regression of the 62 differentially-expressed RBPs.

Gene	OR (95% CI)	*P* value
NXT1	0.002 (0–0.052)	0.006
PELO	0 (0–0.028)	0.019
FAM98A	134.891 (12.223–7503.703)	0.002
YTHDC1	0.009 (0–0.084)	0.001
SPEN	0.005 (0–0.066)	0.002
MEX3D	15.761 (3.807–267.657)	0.006
CTIF	0.001 (0–0.041)	0.012
PAIP2B	0.036 (0.002–0.209)	0.005
PARS2	13.52 (3.664–114.511)	0.002
TIPARP	0.121 (0.025–0.338)	0.001
RNMT	0.035 (0.001–0.21)	0.006
ZMAT3	27.999 (4.929–515.098)	0.003
NOVA1	24.972 (4.49–481.767)	0.005
RIOK3	0.057 (0.005–0.249)	0.003
EZH2	15.864 (3.791–141.354)	0.002
PLD6	0.076 (0.011–0.279)	0.001

ZFP36	0.238 (0.067–0.519)	0.005
PRDX1	292.35 (12.659–47754.741)	0.006
AEN	12.52 (3.305–88.927)	0.002
CTU1	17.521 (3.864–151.39)	0.002
RBM24	0.296 (0.108–0.571)	0.003
PATL1	0.057 (0.006–0.258)	0.002
RPS27	0.138 (0.029–0.397)	0.002
EIF4A3	0.036 (0.002–0.224)	0.004
CLK3	0.096 (0.015–0.343)	0.003
NIP7	12.205 (3.244–83.971)	0.002
IPO13	18.338 (3.702–210.704)	0.004
APOBEC3H	0.466 (0.268–0.702)	0.001
ZCCHC5	4.805 (2.009–17.163)	0.003
EIF4E2	7.687 (2.502–39.611)	0.003
ZC3H12A	0.16 (0.037–0.439)	0.003
NXT2	16.012 (3.44–161.178)	0.004
ERI2	11.737 (3.025–76.228)	0.002
RPP40	5.531 (2.115–21.847)	0.003
CELF2	2.978 (1.585–7.319)	0.004
FDXACB1	5.437 (2.051–22.03)	0.005
CLP1	8.75 (2.711–47.714)	0.002
ENOX1	3.793 (1.712–11.693)	0.005
DNMT3B	3.709 (1.684–10.989)	0.005
DDX25	2.652 (1.454–6.348)	0.009
PABPC4L	3.705 (1.654–11.053)	0.006
MEX3B	1.771 (1.235–2.781)	0.005
TDRKH	3.744 (1.628–11.302)	0.006
CD3EAP	3.398 (1.557–9.86)	0.008
XAB2	0.167 (0.028–0.53)	0.016
PEG10	0.403 (0.184–0.732)	0.009
ERN1	0.212 (0.051–0.576)	0.011
RNASE1	1.36 (1.105–1.754)	0.008
PUS1	3.407 (1.496–10.302)	0.011
MARS2	2.67 (1.377–6.502)	0.012
KHDRBS3	2.311 (1.303–4.897)	0.012
CPSF4L	0.562 (0.354–0.838)	0.008
OAS1	1.567 (1.147–2.292)	0.009
RBM20	0.632 (0.426–0.869)	0.01
DDX28	4.343 (1.635–19.791)	0.018
IFIT1	17.749 (3.721–199.691)	0.003
SBDS	0.453 (0.225–0.789)	0.012
MYEF2	1.534 (1.131–2.201)	0.01
ERN2	1.643 (1.148–2.514)	0.012

PPARGC1B	1.601 (1.129–2.542)	0.019
EXO1	1.997 (1.195–3.84)	0.018
TDRD5	1.81 (1.157–3.322)	0.023

Supplementary Table 4. The CT values of the genes in the clinical samples.

Subject ID	Group	CT value		
		GAPDH	RBM24	RNMT
2022003	Control	14.74525716	18.07840008	20.03488368
2022012	Control	15.84250541	19.18721133	20.05887191
2022008	Control	16.64254397	19.61031053	21.15128554
2022023	Control	15.23590747	20.84740272	19.82626215
2022009	Control	15.01622463	18.46975354	20.61515179
2022002	Control	17.17888197	19.33891721	19.07583418
2022014	Control	14.80743867	17.74364263	21.10228253
2022016	Control	15.99036566	19.33979752	18.0255821
2022018	Control	16.13291468	17.63390788	17.37191675
2022028	Control	15.83299278	19.12467119	19.24682853
OA2022001	OA	17.26160472	21.12238607	21.00300764
OA2022002	OA	16.02803631	20.22529893	22.95781598
OA2022005	OA	15.80973734	20.8112529	21.41373541
OA2022006	OA	15.76713681	18.36677247	19.90915417
OA2022007	OA	15.84871631	21.0072574	22.34982956
OA2022008	OA	14.30135821	20.64377909	22.01430636
OA2022013	OA	15.28747625	21.83092286	20.52466488
OA2022014	OA	16.54314623	21.64015343	21.78957776
OA2022015	OA	15.3648146	23.39388081	21.22732165
OA2022017	OA	16.39616359	20.8044486	21.23231182
OA2022018	OA	17.36021479	20.80605607	21.2718392
